# Evaluation of Personality Profiles in Cluster Headache Patients: A Comparative Analysis with Migraine Patients Using the Minnesota Multiphasic Personality Inventory-3

**DOI:** 10.3390/jcm14186475

**Published:** 2025-09-14

**Authors:** Gerardo Ricardo Zmork-Martínez, Andrea Higuera-Ruiz-de-la-Hermosa, Leonardo Portocarrero-Sánchez, Javier Díaz-de-Terán

**Affiliations:** 1 Department of Neurology, La Paz University Hospital and Stroke Center, 28046 Madrid, Spain; gerardo.zmork@salud.madrid.org (G.R.Z.-M.); anhiguerrh@gmail.com (A.H.-R.-d.-l.-H.); leonardo9493@gmail.com (L.P.-S.); 2 Hospital La Paz Institute for Health Research—IdiPAZ, La Paz University Hospital, Universidad Autónoma de Madrid, 28046 Madrid, Spain

**Keywords:** cluster headache, personality traits, migraine, MMPI-3, psychometrics, chronic pain

## Abstract

**Background/Objectives**: Personality traits in patients with cluster headache (CH) remain understudied compared to migraine patients. This could help improve diagnosis, identify comorbidities, and provide more personalized management of CH. This study aimed to characterize the personality profiles of patients with CH and compare them with those of patients with migraine. **Methods**: This cross-sectional, case–control observational study was conducted at a tertiary hospital’s headache unit (May–August 2024). Patients with CH were compared with migraine patients and healthy controls. Demographic and clinical data were collected, and the Minnesota Multiphasic Personality Inventory-3 (MMPI-3) was administered. **Results**: The study included 28 CH patients (17 with episodic and 11 with chronic CH), 55 migraine patients (34 with episodic migraine and 21 with chronic migraine), and 54 healthy controls. Both patient groups reported significantly more somatic and cognitive complaints than controls (*p* < 0.05). Compared to controls, the migraine group exhibited greater emotional dysfunction, social avoidance, demoralization, introversion, and social anxiety (*p* < 0.05), while the CH group showed greater impulsivity (*p* < 0.05). Directly comparing patient groups, migraine patients displayed greater social avoidance, emotional dysfunction, demoralization, and introversion than the CH group (*p* < 0.05). CH patients also showed a non-significant trend towards behavioral disinhibition, hypomania, a favorable self-image, juvenile conduct problems, substance abuse, and aggressiveness. Patients with CH did not present a higher risk of suicide compared to migraine patients. **Conclusions**: This study identified distinct personality profiles: Migraine patients exhibited greater emotional and interpersonal dysfunction (internal distress and withdrawal), while CH patients exhibited greater externalizing behavioral dysfunction, predominantly involving impulsivity.

## 1. Introduction

Cluster headache (CH) is the most frequent and well-characterized trigeminal-autonomic cephalalgia, with an incidence and prevalence of 2.07/100,000 person-years and 53/100,000 inhabitants, respectively [1]. It is a headache more prevalent in men, although the difference in the male-to-female ratio has been decreasing in recent years to 1.3–2.6:1, likely due to improvements in the diagnosis of previously underdiagnosed women [1,2].

The third edition of the International Classification of Headache Disorders (ICHD-3) bases its diagnosis solely on clinical criteria. CH is characterized by attacks of severe, strictly unilateral headache with durations of 15–180 min. The pain is typically located in the orbital, supraorbital, or temporal region, or a combination of these. Headache attacks are accompanied by ipsilateral trigeminal-autonomic symptoms and/or are associated with restlessness or agitation. The frequency of attacks ranges from one to eight per day [3]. Attacks have a circadian pattern, predominantly occurring at night, and are more common in certain seasons such as spring and autumn [1]. Based on the duration of the headache bouts, CH is classified as episodic, where bouts last from 7 days to one year with remission periods of at least 3 months, or chronic, where bouts last for more than one year with remission periods of less than 3 months [3]. Episodic CH is 6–25 times more frequent than the chronic form, with the latter’s prevalence of the latter being higher in women in Europe and North America than in Asia [4].

The pathophysiology of the disease remains poorly understood, with the trigeminovascular system, autonomic nervous system (via the sphenopalatine ganglion, SPG), and the hypothalamus being proposed as involved structures [1,4]. Given the scarcity of effective treatments, the SPG has emerged as a key therapeutic target. Supporting this, a recent study by Istenič et al. confirmed the feasibility of delivering local anesthetics to the ganglion via a transnasal route, reinforcing its potential in CH management [5].

CH, like migraine, presents a high prevalence of comorbid emotional disorders such as depression and anxiety [6,7,8]. Furthermore, evidence of a correlation between the presence of depression and anxiety and the increased frequency and duration of attacks in these patients has been reported, suggesting that these factors of emotional dysfunctions may facilitate central sensitization and contribute to headache chronicity [9]. In contrast, patients with CH tend to exhibit significant restlessness or agitation during attacks, which includes behaviors such as head rubbing, rocking, pacing, and head banging, among others. This restlessness distinguishes CH from migraine, in which patients often prefer to remain still because movement exacerbates headache pain. This behavior is thought to mitigate or distract from the intense pain caused by headaches, which is considered one of the most severe pain conditions one can experience [1,4,10]. Its high prevalence in these patients has led to its inclusion as a clinical aspect in the diagnostic criteria of the ICHD-3 for CH [3]. Moreover, these patients have a high prevalence of suicidal ideation (55–64%), likely due to the significant impact on quality of life, earning it the name “suicide headache” [1,11,12]. However, rates of attempted and completed suicide are rare, with percentages similar to those of migraine [12,13,14]. Regarding substance use, a high percentage of patients with CH are smokers (60% of patients in the South Korean population) and show a greater tendency towards illicit drug use compared to the general population (31.7% versus 23.8% in the Dutch population) [15,16]. Among illicit drugs, psilocybin, lysergic acid diethylamide (LSD), heroin, amphetamines, and cannabis stand out, indicating a pattern of addictive behavior in patients [16].

Personality refers to is a stable pattern of thought, feeling, and behavior that persists over time and across various situations. It represents an individual’s adaptation to their environment and lifestyle. It has been studied using different theoretical models (the psychobiological model, the Big Five model, or Eysenck’s three-factor model, among others), which share the idea that personality disorders arise from inflexible and rigid patterns. Personality can be viewed on a continuum ranging from good psychological functioning to inflexibility in responding to life’s demands. In this sense, it has been shown that it also influences the chronification of some disorders, such as certain headaches, and may play a role in the clinical management of these patients, potentially improving their response to treatments [17].

Few published studies have investigated the personality of these patients. Graham in 1969 described CH patients as individuals with a weak and dependent personality, who present a hypermasculine external appearance [18]. Two studies that used the Millon Clinical Multiaxial Inventory-III (MCMI-III) questionnaire attempted to characterize the personality profile of these patients, yielding similar findings with some discrepancies. Both studies observed a predominance of obsessive-compulsive traits, coinciding with the presence of other traits, such as histrionic or paranoid traits [6,7]. A recent study analyzed personality traits in 80 patients with CH using the Salamanca questionnaire and compared them with 164 patients with migraine. The researchers observed that the most frequent personality traits in CH were anankastic, anxious, histrionic, schizoid, impulsive, and paranoid traits. When compared with migraine, Cluster A personality traits (schizoid and paranoid) were the most prevalent, although the study was limited by the use of a non-validated screening test [19]. However, another study that compared the personality profiles of 40 patients with CH and 49 migraine patients using the Freiburg Personality Inventory (FPI) found no significant neurotic disturbances, although it did find a greater tendency towards psychosomatic reactions [20]. Several studies suggest that patients with chronic headache show high scores on scales of hypochondria, hysteria, anxiety, depression, and catastrophizing, and these traits have been suggested to influence the chronification process of headaches [17,21]. Despite these prior investigations, inconsistencies in the findings and the use of different psychometric tools highlight the need for further research employing more comprehensive and updated assessments.

This study aimed to characterize the personality profile of patients with CH and compare it with that of patients with migraine. To this end, psychometric evaluations were conducted using the Minnesota Multiphasic Personality Inventory-3 (MMPI-3). This is a self-administered questionnaire with demonstrated reliability for assessing a wide range of psychopathology related to personality disorders. It offers significant psychometric superiority over its predecessors, such as the MMPI-2-RF, by featuring updated 2020 norms and refined scales that address previous limitations. Notably, it introduces new validated scales for compulsivity, impulsivity, and self-importance, enabling a rigorous and quantitative assessment of key clinical hypotheses that older instruments could not adequately measure. The MMPI-3 is a 335-item true/false questionnaire comprising 10 validity scales and 42 substantive scales, with each item answered in a true/false format [22,23]. The present study also aims to contribute to the ongoing adaptation of the MMPI-3 into Spanish in Spain, building on positive results reported in terms of the reliability and validity of the Spanish translation of the scale in the Spanish-speaking population of the United States [24].

## 2. Materials and Methods

A comparative cross-sectional observational study was conducted, comparing patients with CH with patients with migraine and healthy controls. The study was carried out at a tertiary hospital in Madrid (Hospital Universitario La Paz). Patients were recruited from this center’s outpatient headache and general neurology clinics between May and August 2024 after obtaining informed consent. Healthy controls were recruited by approaching patient companions or other healthy individuals present at the hospital.

The inclusion criteria were as follows: patients who met the diagnostic criteria for CH according to the 3rd edition of the ICHD-3, age ≥ 18 years, and accessible electronic or paper medical records [3]. Exclusion criteria included: presence of more than one type of headache, intellectual deficits precluding valid questionnaire completion, those with psychiatric disorders other than anxiety/mood disorders, suicidal behavior, or previously diagnosed personality disorders, inability to complete the test due to not speaking Spanish, and patients with chronic pain related to a disease other than CH and requiring the use of analgesic medication other than that indicated for CH. The control group consisted of volunteers without a diagnosis of CH who met the remaining inclusion criteria and none of the exclusion criteria.

Study participants were also scheduled to complete a Spanish version of the self-administered MMPI-3 questionnaire. Answer sheets were anonymized using a previously assigned code, and relevant sociodemographic data, such as age, sex, education level, and employment status, were also collected. The questionnaires and other materials were used with the permission of Hogrefe TEA Ediciones, S.A.U., the copyright holder for this version of the instrument. The MMPI-3 is an updated version of the MMPI-2-RF and consists of 335 items [23]. The Spanish version provided to patients includes 363 items, 25 more than the standard questionnaire, as this version is an adaptation undergoing final item selection for validation in the Spanish population. It builds upon a version already validated for the US Spanish-speaking population [24,25]. In the questionnaire, answers are marked “V” for true or “F” for false on an optical reading sheet, for subsequent scoring using the electronic scoring systems of Hogrefe TEA.

The MMPI-3 is structured into five groups of scales. First, the Validity Scales, which allow us to determine if the test responses are reliable, include: Combined Inconsistency (CRIN), Variable Response Inconsistency (VRIN), True Response Inconsistency (TRIN), Infrequent Responses (F), Infrequent Psychopathology Responses (Fp), Infrequent Somatic Responses (Fs), Symptom Validity Scale (FBS), Response Bias Scale (RBS), Uncommon Virtues (L), and Adjustment Validity (K).

The following are the Higher-Order Scales, which measure psychological dysfunction from an emotional, cognitive, and behavioral perspective, serving as a starting point for interpreting the other scales of the test: Emotional/Internalizing Dysfunction, Thought Dysfunction, and Behavioral/Externalizing Dysfunction.

The Restructured Clinical Scales allow for a more precise evaluation of the specific dimensions of psychopathology and include Demoralization, Somatic Complaints, Low Positive Emotions, Cynicism, Antisocial Behavior, Ideas of Persecution, Dysfunctional Negative Emotions, Aberrant Experiences, and Hypomanic Activation.

There is also a group of scales called Specific Problem Scales, which, through four subgroups of scales, aim to highlight certain characteristics of interest included in the second-order scales and the restructured clinical scales, where they are not exclusively or detailedly evaluated. These are divided into scales of Somatic/Cognitive Problems (Malaise, Neurological Complaints, Eating Concerns, and Cognitive Complaints), Internalizing Problems (Suicidal Ideation, Helplessness/Hopelessness, Self-Doubt, Inefficacy, Stress, Compulsivity, Worry, Anxiety-Related Experiences, Anger Proneness, and Behavior-Restricting Fears), Externalizing Problems (Family Problems, Juvenile Conduct Problems, Substance Abuse, Impulsivity, Aggression, Activation, and Cynicism), and Interpersonal Problems (Self-Importance, Dominance, Disaffiliativeness, Social Avoidance, and Shyness).

Finally, the questionnaire also includes the Personality Psychopathology Five (PSY-5) Scales, which represent the dimensional model of personality psychopathology by Harkness and McNulty from 1994 and include: Aggressiveness, Psychoticism, Disconstraint, Negative Emotionality/Neuroticism, and Introversion/Low Positive Emotionality [25].

Once the MMPI-3 results were analyzed, patients who showed over-reporting of some symptoms that made the responses highly implausible or were inconsistent were excluded from the study, as these could limit the interpretation of the scales and affect the credibility of the results. Exclusion criteria based on validity scales were: >17 omissions (items i1–i351), a T-score > 79 on VRIN, TRIN, or CRIN, a T-scores > 99 on F or Fp, or other validity scale thresholds being exceeded (Fs ≥ 100, FBS ≥ 90, RBS ≥ 90, L ≥ 80, or K ≥ 80).

Clinical and demographic data were collected anonymously from electronic medical records (Health Care Information System program) or during the in-person interview.

Regarding statistical analysis, quantitative variables were described using means and standard deviations. For qualitative variables, absolute and relative frequencies were presented as percentages. For quantitative variables, the Kolmogorov–Smirnov test (if N > 50) or Shapiro–Wilk test (if N ≤ 50) was used to assess data normality. Clinical and demographic variables were analyzed between the two groups using the Mann–Whitney U test, Student’s *t*-test, and chi-square test (Yates’ correction or Fisher’s exact test depending on the sample size and percentage of individuals in the cells of the contingency table). The age variable was compared between the three groups using the Kruskal–Wallis test, and the sex variable was compared using the Chi-square test (Yates’ correction was applied for samples < 200 patients). To compare personality traits across the three population groups, comparisons were made between the different scores on the MMPI-3 scale for the three groups (CH-migraine, CH-controls, and migraine-controls). As scale scores did not follow a normal distribution in the different groups, the Mann–Whitney U test was used, with the Rank Biserial correlation used as an effect size estimator. Statistical analysis of the data was performed using IBM SPSS Statistics, Version 30 (IBM Corp., Armonk, NY, USA), except for the glass rank biserial correlation coefficient (rrb), which was calculated using Excel spreadsheets from the Z-score obtained with the Mann–Whitney U test calculated with SPSS. All statistical tests were two-tailed, and *p*-values < 0.05 were considered significant.

The study received approval from by the Clinical Research Ethics Committee of the Health Research Institute of Hospital Universitario La Paz (IdiPaz) (approval code PI-6176).

## 3. Results

### 3.1. Sample Characteristics

**Figure 1:**
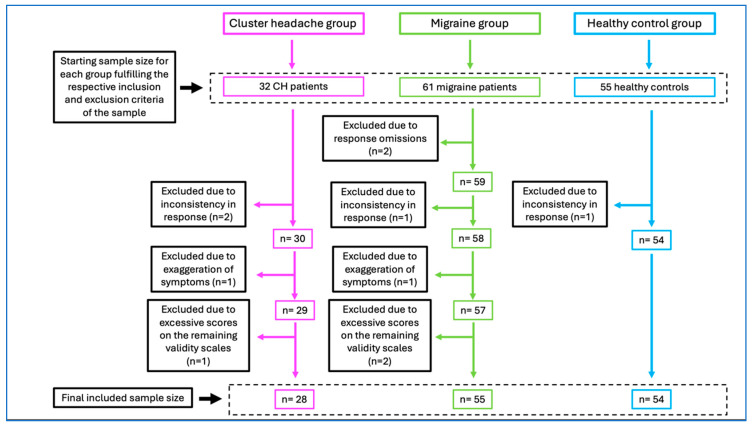
Sample selection process flow chart.

Initially, 32 patients with CH, 61 with migraine, and 55 healthy controls were enrolled in this study. After excluding patients with excessive omissions or elevated T-scores on scales suggesting response inconsistency (VRIN, TRIN, and CRIN), symptom exaggeration (F or Fp), or on other validity scales (Fs, FBS, RBS, L, or K), a final sample of 28 patients with CH, 55 patients with migraine, and 54 healthy controls were retained for analysis. Exclusions were as follows: two migraine patients for omissions; two CH patients, one migraine patient, and one healthy control for response inconsistency; one CH patient and one migraine patient for symptom exaggeration; and one CH patient and two migraine patients for other validity scale elevations (Figure 1).

Within the CH patient group, the mean age was 47.1 ± 13.32 years, with the majority being male (64.3%). In patients with migraine, the mean age was 43 +/− 12.27 years, with the majority being women (81.8%). In the healthy control group, the median age was 35 years (Interquartile Range, IQR: 26–54.25), with 64.8% female participants. The three groups differed significantly in sex (*p* < 0.001) but not statistically significant differences in age (*p* = 0.148). The CH patient group consisted of 17 patients with episodic CH and 11 with chronic CH, whereas the migraine group comprised 34 patients with episodic migraine and 21 with chronic migraine.

### 3.2. Diagnosis-Based Group Comparisons:

#### 3.2.1. Profile of Cluster Headache Patients vs. Healthy Controls

On the validity scales, patients with CH scored significantly (*p* < 0.001) higher on scales related to somatic and/or cognitive symptoms and memory complaints, with a moderate correlation (rrb = 0.42 and 0.47, respectively). Similarly, they showed higher scores for infrequent somatic symptoms (*p* = 0.006), with a moderate correlation (rrb = 0.3). These patients presented themselves in an excessively positive light (*p* = 0.013), a finding with a weak correlation (rrb = 0.27).

Regarding the somatic and cognitive dysfunction scales, patients with CH scored higher than controls on several scales. On the Somatic Complaints and Malaise scales, patients with CH showed statistically significantly higher scores than healthy controls (*p* < 0.001), with moderate correlations (rrb = 0.44 and 0.39, respectively). Furthermore, CH patients also had significantly (*p* = 0.013) higher scores compared to controls on the Cognitive Complaints scale, with a weak correlation (rrb = 0.28). Although not significant, a trend towards slightly higher scores on the scale assessing neurological complaints was observed in CH patients.

Regarding emotional dysfunction scales, patients with CH did not show significant differences compared to healthy controls. However, a non-significant trend towards slightly higher scores was observed in CH patients on the Suicide Ideation, Helplessness/Hopelessness, Compulsivity, and Anger Proneness scales.

Both groups did not show significant differences on the scales specifically assessing thought dysfunction, although a trend was observed for patients with CH towards slightly higher scores on the Aberrant Experiences scale.

On the scales assessing behavioral dysfunction, the only one that showed significant differences (*p* = 0.035) was related to impulsivity, with higher scores in patients with CH than in healthy participants and a weak correlation (rrb = 0.23). For other scales, a non-significant trend towards slightly higher scores was observed on the Behavioral/Externalizing Dysfunction, Juvenile Conduct Problems, Hypomanic Activation, Aggression, and Disconstraint scales. Similarly, a non-significant trend was observed for patients with CH to present slightly lower scores on the Cynicism scale. No trend towards greater substance abuse was evident in patients with CH compared to controls in this study.

Regarding interpersonal functioning, there were no significant differences in the results of the specific scales. Only a non-significant trend for CH patients to score slightly lower on the Introversion/Shyness scale compared to healthy controls was observed.

#### 3.2.2. Profile of Migraine Patients vs. Healthy Controls:

Migraine patients also reported higher levels of somatic and/or cognitive symptoms (*p* < 0.01) and memory complaints (*p* < 0.001). On the somatic and cognitive complaint scales, the migraine group scored significantly (*p* < 0.001) higher scores on the Somatic Complaints and Malaise scales, with a strong correlation (rrb = 0.61 and 0.5, respectively). Neurological Complaints and Cognitive Complaints were also significantly higher in this group (*p* < 0.001 and *p* < 0.01, respectively). The correlation for neurological complaints was moderate (rrb = 0.36), whereas for cognitive complaints was weak (rrb = 0.25). Furthermore, a non-significant trend was observed for migraine patients to present slightly higher scores than healthy controls on the Eating Concerns scale.

Migraine patients showed greater emotional dysfunction than healthy controls, as measured using specific scales. These patients presented statistically significantly (*p* < 0.001) higher scores for the Emotional/Internalizing Dysfunction, Helplessness/Hopelessness, Demoralization, and Low Positive Emotions scales. For all these scales, the correlation coefficients were moderate (0.34, 0.35, 0.32, and 0.34, respectively). The migraine group also showed significantly higher scores on the Suicide Ideation, Self-Doubt, Anxiety-Related Experiences, and Introversion/Low Positive Emotionality scales (*p* = 0.003, 0.006, 0.002, and 0.004, respectively), all with weak correlation coefficients (rrb = 0.29, 0.26, 0.29, and 0.28, respectively). Migraine patients also presented a greater propensity for stress and anger statistically significantly (*p* = 0.023 and 0.017, respectively), with weak correlations (rrb = 0.22 and 0.23, respectively). Although not statistically significant, a trend towards slightly higher scores on the Inefficacy, Dysfunctional Negative Emotions, Worry, and Behavior-Restricting Fears scales was observed in the migraine group compared to healthy controls.

The migraine group also showed a greater tendency to present thought disturbances, with significantly (*p* = 0.009) higher scores on the Aberrant Experiences scale and a weak correlation (rrb = 0.25). This group also presented a higher score on the Thought Dysfunction scale (*p* = 0.027), with a weak correlation (rrb = 0.2). A non-significant trend towards slightly higher scores for psychoticism was also observed in migraine patients.

Regarding behavioral alterations, a statistically significant (*p* = 0.045) lower tendency towards substance abuse was observed in the migraine group, with a weak correlation (rrb = −0.19). On the other hand, these patients showed a non-significant slightly lower tendency towards antisocial behavior and a slightly higher tendency towards aggressive behavior and disconstraint.

Regarding the interpersonal functioning scales, migraine patients scored significantly (*p* = 0.004) higher scores on the Social Avoidance scale, with a weak correlation (rrb = 0.28). They also had significantly (*p* = 0.024) higher scores on the Disaffiliativeness scale than healthy controls, which reflects an avoidance of social situations and a lack of enjoyment in social events. The correlation for this scale was weak (rrb = 0.22). Furthermore, patients with migraine tended to have lower scores on the Self-Importance scale, although this was not statistically significant.

#### 3.2.3. Profile of Cluster Headache Patients vs. Migraine Patients

When comparing the migraine and CH groups, no significant differences were found on the validity scales. There was a non-significant trend for CH patients to present themselves in an excessively positive light and to be more defensive.

The two groups did not show significant differences in terms of somatic and cognitive dysfunction scales, although a trend was observed for migraine patients to present slightly higher scores on the Somatic Complaints, Malaise, Neurological Complaints, and Eating Concerns scales.

Patients with CH presented less emotional dysfunction, with significantly lower scores on the Introversion/Low Positive Emotionality, Emotional/Internalizing Dysfunction, Demoralization, and Low Positive Emotions scales (*p* = 0.015, 0.019, 0.021, and 0.032, respectively). For the first three, the correlations were weak (rrb = −0.27, −0.26, and −0.25). For the Low Positive Emotions scale, the correlation was also weak (rrb = −0.24). For the Stress, Anxiety-Related Experiences, Suicidal Ideation, Helplessness/Hopelessness, Self-Doubt, and Inefficacy scales, which also reflect emotional dysfunction, CH patients had lower, non-significant scores compared to migraine patients.

CH patients also presented less impairment in interpersonal functioning, with significantly lower scores on the Social Avoidance and Introversion/Shyness scales (*p* = 0.008 and *p* = 0.049, respectively). The correlation was weak in both cases (rrb = −0.29 and −0.22, respectively). A non-significant trend was also observed for CH patients towards high scores on the Self-Importance and Aggressiveness scales. Similarly, migraine patients had a non-significant greater tendency towards higher scores on the Disaffiliativeness scale.

Both groups showed no differences in terms of thought dysfunction scales, although a non-significant trend was observed for migraine patients to score slightly higher on the Psychoticism scale.

Regarding behavioral dysfunction scales, there were no significant differences between the two groups, but a greater tendency was observed in CH patients towards slightly higher scores on the Behavioral/Externalizing Dysfunction, Hypomanic Activation, Juvenile Conduct Problems, Substance Abuse, Impulsivity, Activation, and Disconstraint scales. However, migraine patients showed a tendency towards slightly higher scores on the Family Problems and Cynicism scales.

Table 1 and Table 2 present the normalized MMPI-3 item score differences observed between the CH, migraine, and matched healthy control groups.

## 4. Discussion

Attempts to characterize personality profiles in CH patients date back to the 20th century [18,26,27,28]. Initially, Graham described them as ambitious and hardworking individuals with a strong sense of upward social mobility. He also noted that these patients conceal a personality with significant dependency needs, especially towards an authority figure, beneath a hypermasculine facade. He famously termed this the “leonine mouse syndrome” or referred to them as “Mouse living inside lions” [27,28]. He commented that men are often accompanied to the doctor by their wives, who request prescriptions and reports and manage appointments for them. They also tend to harbor internal feelings of great anger, guilt, and inadequacy and are reluctant to disclose them to strangers. He also described histrionic traits and frequent evidence of underlying depression [26,27].

Two studies using the MCMI-III to assess personality traits in patients with CH, including 26 and 56 patients, respectively, both reported a predominance of compulsive traits, followed by histrionic, narcissistic, paranoid, schizoid, and avoidant traits [6,7]. A recent study comparing the personality traits of patients with migraine observed similar traits in the CH group to those previously reported, with a predominance of an obsessive-compulsive profile and other traits, such as impulsivity [19]. In a study by Telesca et al., which included 56 patients with CH, 87% of their patient sample presented with personality dysfunctions, and 57% had persistent personality traits of clinical concern. Furthermore, they identified two patterns of personality functioning in patients with CH: psychological dysregulation and social engagement. The psychological dysregulation dimension stood out in these patients because of the presence of negativistic, sadistic-aggressive, borderline, and compulsive traits, while the social engagement dimension was characterized by the presence of narcissistic, histrionic, avoidant, and schizoid traits. The latter dimension appears to be more related to educational level and youth [6].

The data obtained in our study suggest that these patients, compared with healthy controls, showed a greater tendency towards somatic and cognitive dysfunction. Overall, elevated scores on these scales reflect feelings of general weakness and poor health perception. Furthermore, an elevated score on the specific scale for cognitive complaints indicates problems with concentration, memory complaints, and difficulty coping with stress, as well as low frustration tolerance in these patients. Previous studies have also reported similar findings, with patients describing a poor or very poor perception of health compared to healthy controls (9% vs. 1%, respectively) [1]. In addition, these patients present elevated scores on the Headache Impact Test-6 (HIT-6) and 36-Item Short Form Health Survey (SF-36) scales, which indicate the significant impact this headache has on their health and quality of life [9]. Rozen et al. found that approximately 20% of patients with CH lost their jobs due to headaches, and an additional 8% were not working or were disabled by headaches [12].

Patients with CH in our sample showed a greater tendency to present an excessively positive self-image compared to controls, which could align with the facade described by Graham, whereby these patients conceal negative aspects of themselves (“the leonine mouse syndrome”).

On other scales where we found statistically significant differences, we found a greater tendency towards impulsivity, a finding consistent with previous studies [19]. Although no statistically significant differences were found for other scales of behavioral dysfunction, a trend towards higher scores was observed on some of the scales that describe a greater tendency towards disinhibition, externalizing behaviors, slightly greater aggressiveness, juvenile conduct problems, or hypomanic traits. This latter scale is associated with the feeling described by patients of increased restlessness and motor agitation, which has been considered a distinctive feature of these patients (present in up to 80–90% of patients) and differentiates them from migraine [29]. It should be emphasized that hypothalamic dysfunction, likely ventromedial, is also hypothesized to explain both symptoms [4]. These patients have a greater tendency towards aggressive behavior, which can become violent and even self-destructive during attacks [12,28,29]. These data are also supported by previous studies, where a greater tendency of these patients to present histrionic traits has been observed, which may be reflected in this test by the subscales of self-importance, activation, impulsivity, and hypomanic activation [6,7,19]. Notably, no differences were observed in our sample of patients with CH compared to controls in substance use, in contrast to several previous studies that report these patients have a greater tendency to consume licit and illicit drugs compared to the general population [14,16,30,31]. De-Coo et al. observed in a sample of 756 individuals who completed a questionnaire a greater lifetime use of illicit drugs in CH patients compared to controls (31.7% vs. 23.8%), being higher in men than in women [16]. A prevalence of 0.9% of substance use disorder in patients with CH versus 0.1% in the general population has also been suggested; however, the data are limited [32].

Studies suggest that CH patients have a 3–5.6 times higher risk of depression compared to controls, especially in the more active phases of the disease, and this association may be influenced by the presence of sleep disorders [33,34]. Furthermore, some studies support the increased risk of depression, with CH patients also presenting significantly higher levels of depression and anxiety measured by the Hospital Anxiety and Depression Scale (HADS), the Beck Depression Inventory and the State-Trait Anxiety Inventory [9,33,35,36,37]. Based on the HADS, approximately 52.73–75.7% of patients with CH suffer from anxiety and 43–47.27% suffer from depression [35,36]. Therefore, the limited emotional dysfunction observed in our sample compared with what would be expected based on these reports is remarkable. This was despite our observation of non-significant trends on scales related to suicide, compulsivity, anger proneness, hopelessness, or helplessness. The literature mentions a higher suicidal risk (especially during the ictal period) [1,12,38]; therefore, it is noteworthy that despite the described trend, the difference was not more pronounced in the present study. This could partly be explained by the absence of differences in the scores on the demoralization scale in either group. Demoralization reflects a profound sense of hopelessness, inability to manage problems, and a feeling of being trapped, which has been suggested as an even stronger predictor of suicidal ideation than depression [39]. The tendency towards compulsivity related to obsessive-compulsive traits has also been described by several recent studies, which report it as one of the most frequent characteristics of these patients [6,7,19]. A non-significant higher score was also observed on the Aberrant Experiences scale related to disorganized thinking, as evidenced in other studies [7].

Kudrow et al. (1974) compared 20 patients with CH with 31 healthy controls and found that patients with CH are more extroverted, affectionate, conscientious, responsible, and respectful of rules [40]. This may coincide with the trend observed in our study, although not statistically significant, for patients to be trustworthy or well-intentioned by presenting lower scores on the cynicism scale and less shyness and introversion.

Historically, migraine patients have been initially described as orderly, perfectionistic, inflexible, and prone to overreacting to problems. Various studies have shown heterogeneity in the expression of personality traits in these patients, although most studies have shown a greater tendency towards neuroticism [41,42,43,44,45]. Some of these studies are based on models such as Eysenck’s three factors or the Big Five. Within the latter model, it has also been shown that they tend to be more responsible and agreeable. Other variables may include harm avoidance, persistence, or self-directedness (psychobiological model), or extraversion and psychoticism (Eysenck’s three-factor model). In relation to the above, a meta-analysis by Garramone et al. in 2020 [41] found a greater tendency towards harm avoidance, persistence, and lower self-directedness, and on the other hand, a higher level of neuroticism and a lower level of extraversion based on these models. High levels of neuroticism were associated with more severe depression symptoms in these patients. The authors suggested that patients with migraine are prone to a pessimistic view regarding the anticipation of future problems and exhibit passive avoidance behaviors, such as fear of uncertainty and rapid fatigability [41]. Some studies have also linked higher neuroticism and lower extraversion to a greater likelihood of developing headaches. Similarly, neuroticism has been linked to behaviors such as greater substance use, sleep disorders (including bruxism), a higher risk of obesity, and possibly a higher risk of developing dementia. In contrast, being less extroverted could be more strongly associated with greater stress and poorer sleep quality, as well as a greater likelihood of being overweight [46].

In our migraine sample, the most prominent finding was a higher level of somatic and cognitive complaints than in healthy controls. This was followed by high levels of emotional dysfunction, with high scores on scales related to mood or affective state, helplessness or hopelessness, demoralization, and low positive emotions. These results are similar to those of other studies [41,42,43,44,45,46].

Significant, though with a smaller effect size, were findings of a greater tendency towards suicide, lack of self-confidence or feelings of worthlessness, anxiety, social disinterest and anhedonia, as well as a greater propensity for stress and anger. However, there is evidence that people with migraine can control anger more than controls or CH patients [47]. Furthermore, the patients presented a significantly greater tendency towards disorganized thinking and, at the behavioral level, a greater tendency towards substance use. Regarding interpersonal functioning, patients with migraine appeared to avoid social situations and lacked enjoyment of social events and activities. These patients present a profile of social withdrawal. This last point has been described in previous studies, such as Huber et al., in which the first version of the MMPI was used [41,44,48].

Muñoz et al. compared 80 CH patients (83% episodic CH) with 164 migraine patients using the Salamanca questionnaire in the absence of a healthy control group. Despite being limited by the use of a non-validated personality test, they observed a higher frequency of anankastic (52.5%), anxious (47.5%), histrionic (45%), schizoid (42.5%), impulsive (32.5%), and paranoid (30%) personality traits in patients with CH. Patients with CH showed a statistically significantly higher prevalence of paranoid and schizoid traits than those with migraine. Although they did not reach statistical significance, a tendency was also found for patients with CH to have a higher prevalence of anankastic, histrionic, schizotypal, and impulsive traits, while migraine patients had a slightly higher percentage of dependent and anxious traits [19]. A previous study from 1981 by Cuypers et al. also found no significant differences using the FPI when comparing 40 patients with CH and 49 migraine patients [20].

In our study, when comparing both groups, a markedly lower level of emotional dysfunction was observed in patients with CH than in those with migraine. Specifically, patients with migraine seemed to have greater social disinterest and anhedonia, mood and affective alterations, demoralization (general unhappiness and dissatisfaction), and a scarcity of positive emotions. There were also no differences in the stress and anxiety scales, although the trend was greater in patients with migraine. These results are consistent with the trends observed by Muñoz et al. [19]. Although Rausa et al. reported a greater tendency towards anger in patients with CH than in those with migraine, we did not find any differences between the two groups [47]. Our migraine patients had a non-significant tendency to have low self-confidence, be more indecisive, present feelings of hopelessness, or have greater suicidal ideation compared to patients with CH. This last point is striking, as studies suggest that suicidal ideation is higher in patients with CH [49]. However, more recent studies have shown disparate data with possibly greater suicidal ideation in patients with CH, with similar percentages of suicide attempts in both groups [12,13,14].

Patients with CH, compared to those with migraine, appeared less avoidant of social situations and showed greater enjoyment of social events and activities, and presented with less social anxiety. Furthermore, although not significant, patients with CH had a greater tendency towards self-importance (seeking recognition and feeling superior) and aggressiveness.

We did not find significant behavioral alterations, but patients did show a greater tendency towards externalizing behaviors, hypomanic activation, juvenile conduct problems, substance abuse, a high level of activation or energy, and disinhibition, while patients with migraine showed a greater tendency to have conflicting family relationships and distrust of others.

Therefore, these data reinforce the idea that there are different personality profiles between patients with CH and migraine Further study of these traits in these and other headaches could help guide the differential diagnosis between different headaches, which is important in a pathology with an average diagnostic delay of five or more years [1,12].

As mentioned in this study, many of these psychological factors can influence the risk of CH chronification [11]. Adequately addressing these symptoms could, therefore, have a positive impact on health care costs by reducing the need for care and medication [2,13]. It would also reduce indirect costs due to absenteeism, as chronic forms of CH receive five times more disability pensions than episodic forms [1,2]. In addition, it would improve the quality of life and reduce the burden, fragility, and risk of suicidality in these patients [2,13].

Knowing the personality profiles of these patients could also help personalize their management. First, adapting the “soft skills” of the consultation, such as communication, to the personality of each patient can improve patient care. They can also help determine whether there are patients who benefit from psychotherapy, such as behavioral and other non-pharmacological therapies, although data on the efficacy of such approaches, including cognitive-behavioral therapy, remain scarce [7,11,50]. Considering the significant impact these behavioral symptoms have on quality of life during ictal and interictal periods, a formal evaluation of targeted therapeutic approaches is warranted. This includes not only psychotherapies but also interventional procedures like SPG stimulation and occipital nerve stimulation, which represents a promising approach for managing CH [50].

Furthermore, there is evidence that personality can influence pharmacological responses in patients with headache [17]. For example, in patients with migraine, personality traits have been shown to act as predictive factors for response to certain preventive treatments, such as Onabotulinumtoxin A or monoclonal antibodies against Calcitonin Gene-Related Peptide (CGRP). In the case of Onabotulinumtoxin A results showed that dependent personality was associated with a poorer response. Similarly, it was found that with Erenumab, Cluster C personality disorders, studied as a composite variable, and the presence of serious life events are predictors of poor response [51,52]. However, in a study that included three different anti-CGRP mAbs, non-responding patients showed a greater tendency towards a depressive state and anhedonia [53].

Another important aspect of the personalized management of these patients is the choice of preventive medication that best addresses the profile of associated emotional and behavioral symptoms or psychiatric comorbidity. For example, mood-stabilizing drugs, such as lithium or valproic acid, should be considered for patients with manic symptoms. Special care must also be taken with drug interactions, as the combination of serotonergic antidepressants with preventives, such as lithium, could induce the risk of serotonin syndrome. This underscores the importance of collaboration between different specialists (neurologists, psychologists, psychiatrists, primary care physicians, etc.) to ensure the safety and efficacy of interventions is also deduced [54].

Our study had some limitations that should be highlighted. First, the sample size was small in this study. Although this was a single-center study, our hospital is a national referral center for patients with CH, and more than half of the patients we manage are referred from other hospitals across Spain. In addition, the cross-sectional nature of the study precludes the establishment of causal relationships. Furthermore, the fact that our sample of patients with CH had a higher percentage of women (35.7%) than other published series may limit the comparability of our results with those of other studies. This could explain the higher percentage of chronic CH or the lower consumption of licit or illicit drugs in our study [4,19]. However, the proportion of women and men in our study is in line with the estimated prevalence, which accounts for previous underdiagnosis in this subgroup of patients [1,2]. The sex distribution among the three groups was unequal, although expected, given that migraine has a clear predominance in women. In addition, the predominance of chronic forms in both groups (60.7% in patients with CH and 61.8% in patients with migraine) may have influenced the results of our study, which may have differed in some aspects from other reported studies. Therefore, it would be interesting to evaluate the differences in personality profiles between patients with episodic and chronic CH in future studies to confirm this hypothesis.

## 5. Conclusions

Our findings reinforce the idea that distinct personality profiles exist in patients with CH and migraine. The CH profile in our study is characterized by present greater impulsivity and, although not significant, a trend towards a pattern of externalizing behaviors suggestive of histrionic traits (including a more favorable self-image, hypomania, aggressiveness, and disinhibition), juvenile conduct problems, and substance abuse. In contrast, the migraine profile is characterized by greater emotional dysfunction, social avoidance, demoralization, introversion, and social anxiety, with non-significant trends towards greater anxiety and stress. Contrary to some reports in the literature, our patients with CH did not present a higher risk of suicide than those with migraine.

Further research on the personality profiles of patients with primary headaches, such as CH or migraine, is warranted, as a deeper understanding could improve patients care, guide clinical diagnosis, and provide more personalized management. In this regard, the personality characteristics of these patients may have predictive and prognostic value, which may help in the future selection of both non-pharmacological therapies, such as certain psychotherapies, and pharmacological therapies that best suit the profile of each patient.

## Figures and Tables

**Table 1:** Normalized MMPI-3 item score differences: comparisons of cluster headache and migraine groups with healthy controls.

Normalized Scales	Control-Migraine		Control-Cluster Headache	
	rrb ^1^	*p*	rrb	*p*
**Combined Response Inconsistency**	−0.14	0.157	−0.07	0.523
**Variable Response Inconsistency**	−0.09	0.326	−0.02	0.827
**True Response Inconsistency**	−0.12	0.214	−0.1	0.348
**Infrequent Responses**	–0.26	0.008	−0.2	0.067
**Infrequent Psychopathology Responses**	−0.06	0.527	−0.04	0.719
**Infrequent Somatic Responses**	−0.39	<0.001	−0.3	0.006
**Symptom Validity Scale**	−0.62	<0.001	−0.42	<0.001
**Response Bias Scale**	−0.48	<0.001	−0.47	<0.001
**Uncommon Virtues**	−0.13	0.175	−0.27	0.013
**Adjustment Validity**	0.18	0.062	0.06	0.588
**Emotional/Internalizing Dysfunction**	−0.34	<0.001	−0.07	0.537
**Thought Dysfunction**	−0.21	0.027	−0.13	0.235
**Behavioral/Externalizing Dysfunction**	0.05	0.566	−0.12	0.261
**Demoralization**	−0.32	<0.001	−0.06	0.6
**Somatic Complaints**	−0.61	<0.001	−0.44	<0.001
**Low Positive Emotions**	−0.34	<0.001	−0.07	0.499
**Antisocial Behavior**	0.12	0.196	0.01	0.925
**Ideas of Persecution**	−0.07	0.461	−0.02	0.867
**Dysfunctional Negative Emotions**	−0.12	0.228	−0.06	0.596
**Aberrant Experiences**	−0.25	0.009	−0.18	0.096
**Hypomanic Activation**	0.01	0.898	−0.18	0.107
**Malaise**	−0.51	<0.001	−0.39	<0.001
**Neurological Complaints**	−0.36	<0.001	−0.19	0.087
**Eating Concerns**	−0.14	0.136	−0.01	0.936
**Cognitive Complaints**	−0.25	0.010	−0.28	0.013
**Suicidal Ideation**	−0.29	0.003	−0.17	0.128
**Helplessness/Hopelessness**	−0.35	<0.001	−0.19	0.08
**Self-Doubt**	−0.26	0.006	−0.08	0.47
**Inefficacy**	−0.12	0.193	0.04	0.719
**Stress**	−0.22	0.023	−0.08	0.478
**Worry**	−0.18	0.066	−0.08	0.477
**Compulsivity**	−0.05	0.608	−0.12	0.269
**Anxiety-Related Experiences**	−0.29	0.002	−0.08	0.478
**Anger Proneness**	−0.23	0.017	−0.17	0.13
**Behavior-Restricting Fears**	−0.13	0.166	−0.05	0.62
**Family Problems**	−0.08	0.387	0.07	0.535
**Juvenile Conduct Problems**	0.08	0.400	−0.14	0.21
**Substance Abuse**	0.19	0.045	0.03	0.766
**Impulsivity**	−0.07	0.441	−0.23	0.035
**Activation**	0.04	0.640	−0.08	0.493
**Aggression**	−0.12	0.198	−0.11	0.298
**Cynicism**	0.01	0.920	0.17	0.117
**Self-Importance**	0.1	0.298	−0.09	0.42
**Dominance**	0.06	0.558	−0.01	0.902
**Disaffiliativeness**	−0.22	0.024	−0.05	0.662
**Social Avoidance**	−0.28	0.004	0.05	0.624
**Shyness**	−0.09	0.341	0.15	0.186
**Aggressiveness**	−0.04	0.643	−0.08	0.469
**Psychoticism**	−0.16	0.105	−0.03	0.778
**Disconstraint**	−0.11	0.259	−0.12	0.261
**Negative Emotionality/Neuroticism**	−0.16	0.103	−0.05	0.648
**Introversion/Low Positive Emotionality**	−0.28	0.004	0.01	0.949

Abbreviations: ^1^ rrb, glass rank biserial correlation coefficient.

**Table 2:** Normalized MMPI-3 Item score differences in the comparison between cluster headache and migraine.

Normalized Scales	Migraine-Cluster Headache	
	rrb ^1^	*p*
**Combined Response Inconsistency**	0.05	0.635
**Variable Response Inconsistency**	0.06	0.569
**True Response Inconsistency**	−0.01	0.945
**Infrequent Responses**	0.09	0.435
**Infrequent Psychopathology Responses**	0.02	0.868
**Infrequent Somatic Responses**	0.16	0.146
**Symptom Validity Scale**	0.19	0.091
**Response Bias Scale**	0.07	0.514
**Uncommon Virtues**	−0.16	0.147
**Adjustment Validity**	−0.12	0.266
**Emotional/Internalizing Dysfunction**	0.26	0.019
**Thought Dysfunction**	0.1	0.375
**Behavioral/Externalizing Dysfunction**	−0.13	0.226
**Demoralization**	0.25	0.021
**Somatic Complaints**	0.21	0.057
**Low Positive Emotions**	0.24	0.032
**Antisocial Behavior**	−0.04	0.737
**Ideas of Persecution**	0.06	0.598
**Dysfunctional Negative Emotions**	0.05	0.629
**Aberrant Experiences**	0.07	0.534
**Hypomanic Activation**	−0.2	0.069
**Malaise**	0.17	0.119
**Neurological Complaints**	0.19	0.076
**Eating Concerns**	0.12	0.294
**Cognitive Complaints**	−0.01	0.946
**Suicidal Ideation**	0.13	0.231
**Helplessness/Hopelessness**	0.15	0.163
**Self-Doubt**	0.2	0.069
**Inefficacy**	0.15	0.16
**Stress**	0.13	0.252
**Worry**	0.07	0.543
**Compulsivity**	−0.08	0.469
**Anxiety-Related Experiences**	0.17	0.113
**Anger Proneness**	0.02	0.824
**Behavior-Restricting Fears**	0.1	0.377
**Family Problems**	0.15	0.166
**Juvenile Conduct Problems**	−0.2	0.066
**Substance Abuse**	−0.12	0.292
**Impulsivity**	−0.17	0.128
**Activation**	−0.12	0.282
**Aggression**	−0.02	0.843
**Cynicism**	0.14	0.19
**Self-Importance**	−0.19	0.088
**Dominance**	−0.06	0.569
**Disaffiliativeness**	0.15	0.16
**Social Avoidance**	0.29	0.008
**Shyness**	0.22	0.049
**Aggressiveness**	−0.12	0.261
**Psychoticism**	0.12	0.264
**Disconstraint**	−0.2	0.069
**Negative Emotionality/Neuroticism**	0.07	0.527
**Introversion/Low Positive Emotionality**	0.27	0.015

Abbreviations: ^1^ rrb, glass rank biserial correlation coefficient.

## Data Availability

The original contributions presented in this study are included in the article. Further inquiries can be directed to the corresponding authors.

## Abbreviations

The following abbreviations are used in this manuscript:

CGRP	Calcitonin Gene-Related Peptide
CH	Cluster Headache
F	Infrequent Responses
FBS	Symptom Validity Scale
Fp	Infrequent Psychopathology Responses
FPI	Freiburg Personality Inventory
Fs	Infrequent Somatic Responses
HADS	Hospital Anxiety and Depression Scale
HIT-6	Headache Impact Test-6
ICHD-3	International Classification of Headache Disorders
K	Adjustment Validity
L	Uncommon Virtues
LSD	Lysergic acid diethylamide
MCMI-III	Millon Clinical Multiaxial Inventory-III
MMPI	Minnesota Multiphasic Personality Inventory
MMPI-2-RF	Minnesota Multiphasic Personality Inventory-2-RF
MMPI-3	Minnesota Multiphasic Personality Inventory-3
RBS	Response Bias Scale
Rrb	Glass Rank Biserial Correlation Coefficient
SF-36	36-Item Short Form Health Survey
SPG	Sphenopalatine ganglion
TRIN	True Response Inconsistency
VRIN	Variable Response Inconsistency
